# A Re-Evaluation of the Relationship between Morphology and Pathogenicity in *Candida* Species

**DOI:** 10.3390/jof6010013

**Published:** 2020-01-13

**Authors:** David Kadosh, Vasanthakrishna Mundodi

**Affiliations:** Department of Microbiology, Immunology and Molecular Genetics, University of Texas Health Science Center at San Antonio, 7703 Floyd Curl Dr., MC: 7758, San Antonio, TX 78229, USA; Mundodi@uthscsa.edu

**Keywords:** *Candida* species, morphology, pathogenesis, yeast, filaments

## Abstract

Many pathogenic *Candida* species possess the ability to undergo a reversible morphological transition from yeast to filamentous cells. In *Candida albicans*, the most frequently isolated human fungal pathogen, multiple lines of evidence strongly suggest that this transition is associated with virulence and pathogenicity. While it has generally been assumed that non-*albicans Candida* species (NACS) are less pathogenic than *C. albicans*, in part, because they do not filament as well, definitive evidence is lacking. Interestingly, however, a recent study suggests that filamentation of NACS is associated with reduced, rather than increased, pathogenicity. These findings, in turn, challenge conventional views and suggest that there are fundamental evolutionary differences in the morphology–pathogenicity relationship in *C. albicans* vs. NACS. The findings also raise many new and intriguing questions and open new avenues for future research, which are discussed.

## 1. Introduction

*Candida* species represent the fourth leading cause of hospital-acquired bloodstream infections in the U.S. [1,2]. In healthy individuals, these species are typically part of the normal human microbiota, residing on mucosal surfaces [3,4,5]. However, *Candida* species can also cause systemic infections and colonize internal organs in a variety of immunocompromised patients, as well as individuals who have received surgical implants [6,7,8]. Sepsis caused by *Candida albicans* has an associated mortality rate of about 40%, which is higher than that of any bacterial sepsis [2,9,10]. In the U.S. alone, approximately $1 billion per year is spent on the treatment of patients with hospital-acquired *Candida* infections [11].

Approximately 50% of all *Candida* infections can be attributed to *C. albicans*, the most frequently isolated human fungal pathogen, while the remainder are caused by the less pathogenic non-*albicans Candida* species (NACS) [12]. However, infections by NACS are on the rise and *Candida glabrata*, *Candida tropicalis*, *Candida dubliniensis*, *Candida parapsilosis*, *Candida auris,* and *Candida krusei* are considered as emerging opportunistic pathogens [13,14]. Of greater concern, several of these species show increased resistance to antifungal drugs [13,15] and have been added to the Centers for Disease Control (CDC) antibiotic resistance threat list (https://www.cdc.gov/drugresistance/biggest-threats.html#candida). Candida species are also known to possess a variety of properties that have typically been associated with pathogenesis, including the ability to adhere to host cells, form biofilms, secrete degradative enzymes, switch phenotypes, and undergo a morphological transition [4,5,16,17,18].

## 2. Morphogenesis in *Candida* Species

*Candida* species can typically grow in either the yeast or filamentous form. Yeasts are single oval-shaped budding cells, whereas filaments consist of elongated cells attached end-to-end [19,20]. There are two types of filamentous morphologies: pseudohyphae and hyphae. Pseudohyphae are elliptical in shape, have constrictions at cell junctions, and are typically highly branched. In contrast, hyphae possess parallel-sided cell walls, true septa (lacking constrictions), and generally show less branching [19]. In addition, in hyphae, the first nuclear division occurs in the initial germ tube, whereas in yeast and pseudohyphal cells, this event occurs across the mother cell–bud neck [21,22,23]. 

It is important to note that not all *Candida* species can grow in all three morphologies, and the proportion of cells that grow in a particular morphology can vary among *Candida* species. For example, *C. glabrata* grows almost exclusively in the yeast morphology, whereas *C. albicans* and *C. tropicalis* can be found in all three morphologies. Other species such as *C. parapsilosis* and *Candida guilliermondii* are only found in yeast and pseudohyphal morphologies but grow predominantly in the yeast form [16,20,24].

A fourth morphology, chlamydospores, can be found in several *Candida* species, including *C. albicans* and *C. dubliniensis*. Chlamydospores are thick-walled, large, round cells. They typically form at the ends of hyphal suspensor cells in response to nutrient starvation conditions and are rarely observed in infected tissues [25,26]. 

## 3. Relationship between Morphology and Pathogenicity in *C. albicans*

A large majority of previous studies have suggested that there is a strong correlation between the ability of *C. albicans* to undergo a reversible yeast–filament transition and virulence. Studies carried out on clinical isolates have shown a clear correlation between the invasion of reconstituted oral mucosal surfaces and an increased number of hyphal filaments [27]. In addition, early work showed that *C. albicans* strains locked in either the yeast or filamentous form were highly attenuated for virulence in a mouse model of systemic candidiasis [28,29,30]. A subsequent study provided more convincing evidence and showed that allowing a yeast-locked strain to transition to filaments at various time points during the course of a systemic infection was sufficient to promote virulence [31]. More specifically, *NRG1*, a strong transcriptional repressor of *C. albicans* filamentation, was placed under control of the *E. coli tet* operator in a strain constitutively expressing a *tetR*-transactivator fusion protein. In the absence of doxycycline (Dox), *NRG1* was expressed at constitutively high levels, locking *C. albicans* in the yeast form and resulting in highly attenuated virulence. In the presence of Dox, *NRG1* was shut off, allowing cells to transition to filaments in response to growth at 37 °C. Importantly, regulation of *NRG1* expression could be controlled during a mouse systemic infection in vivo simply by the presence or absence of Dox in the drinking water. Using this system, Saville et al. found that the addition of Dox to the drinking water at various post-infection time points was sufficient to promote virulence, thus providing strong evidence that the *C. albicans* yeast–filament transition is required for pathogenesis [31]. A subsequent converse study demonstrated that expression of *UME6*, a key filament-specific transcription factor, under control of the *E. coli tet* operator, could drive strong hyphal growth as well as promote tissue invasion and pathogenicity in both a murine model of systemic candidiasis and 3-dimensional reconstituted model of the human oral epithelium [32,33].

One caveat to the experiments described above is that morphological effects were generated by deletion or overexpression of transcriptional regulators that control large sets of target genes. Therefore, it cannot be excluded that attenuated virulence is observed as a consequence of misexpression or overexpression of target genes that are involved in other virulence-related processes, rather than filamentation per se. However, an elegant study by the Wang laboratory has demonstrated that a *C. albicans* strain mutated for *HGC1*, which encodes a cyclin-related protein important for septin phosphorylation, is defective for filamentation and highly attenuated for virulence in a mouse model of disseminated candidiasis [34]. Importantly, the *hgc1*Δ/Δ mutant did not affect the expression of filament-specific genes, suggesting that the virulence defect was specifically due to reduced filamentation ability. In addition, a more recent large-scale functional genomic study has shown a general correlation between *C. albicans* mutants that are defective for filamentation and those that are attenuated for virulence [35]. However, a previous screen identified several mutants with defects in kidney infectivity that showed normal morphology, suggesting a more complex relationship between these processes [36].

## 4. Relationship between Morphology and Pathogenicity in Non-*albicans Candida* Species

Non-*albicans Candida* species are believed to be less pathogenic than *C. albicans* for a variety of reasons, including an increased sensitivity to host environmental stresses and reduced ability to form biofilms, adhere to host cells, and secrete a variety of degradative enzymes [16,37]. In addition, non-*albicans Candida* species do not filament as readily or robustly as *C. albicans*. For example, while *C. albicans* shows strong filament induction in response to a variety of host environmental cues, including serum and temperature, neutral/alkaline pH, nitrogen starvation, high CO_2_/O_2_ ratio, certain carbon sources and several human hormones (including progesterone, chorionic gonadotrophin, estradiol and leutinizing hormone), non-*albicans Candida* species such as *C. tropicalis*, *C. parapsilosis* and *C. guilliermondii* filament in response to very limited and defined growth conditions [4,37,38,39,40,41,42,43]. In addition, certain key transcriptional events associated with *C. albicans* filamentation appear to be only partially conserved in NACS [43]. For example, while *UME6* is strongly induced during *C. albicans* filamentation, orthologs of *UME6* are induced at lower levels, and in some cases with delayed timing, in *C. tropicalis*, *C. parapsilosis* and *C. guilliermondii*. Induction of *UME6* orthologs in *C. tropicalis* and *C. parapsilosis* has been shown to drive strong filamentation and biofilm formation as well as expression of several (but not all) orthologs of *C. albicans* filament-specific genes in these species. *NRG1* appears to have retained its function as a repressor of filamentation in *C. tropicalis* and *C. parapsilosis* but not *C. guillermondii*. Interestingly, however, unlike the situation in *C. albicans*, *NRG1* transcript levels do not appear to be down-regulated in these species in response to filament-inducing conditions [43]. 

Based on the studies described above, as well as the strong correlation between filamentation and virulence in *C. albicans*, the reduced filamentation ability of NACS was expected to be an important contributor to the decreased pathogenicity of these species. Indeed, two initial in vitro studies indicated that *C. tropicalis* clinical isolates with increased filamentation ability showed enhanced invasion of a reconstituted human oral epithelial cell layer as well as increased epithelial cell damage [44,45]. In addition, a study using the mouse model of systemic candidiasis showed that strongly filamentous *C. tropicalis* clinical isolates demonstrated slight increases in virulence and tissue invasion, although this study was carried out with immunosuppressed mice [46]. However, previous studies have shown that the ability of *C. parapsilosis* isolates to form pseudohyphae does not correlate with the ability to invade in a reconstituted oral epithelium model and that a hyperfilamentous *C. parapsilosis* mutant is attenuated for pathogenicity in both *Galleria* and mouse models of candidiasis [47,48]. 

**Figure 1:**
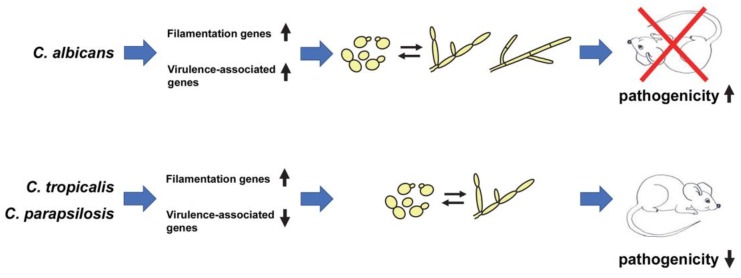
Model for evolutionary differences in the relationship between morphology and pathogenicity among *Candida* species. In *C. albicans*, genes important for filamentation are coordinately expressed with those involved in other virulence-related processes, leading to a strong association of the yeast–filament transition with pathogenicity. In *C. tropicalis* and *C. parapsilosis*, genes involved in filamentation are also induced but there is an accompanying down-regulation of certain genes involved in virulence-related processes, resulting in an inverse correlation between filamentation and pathogenicity. For second column, upward and downward arrows indicate increased and decreased gene expression, respectively. For fourth column, upward and downward arrows indicate increased and decreased pathogenicity, respectively. Please note that not all virulence-related genes may be down-regulated during filamentation of NACS and that alternative mechanisms not associated with changes in gene expression may account for the reduced pathogenicity of *C. tropicalis* and *C. parapsilosis* upon filamentation. Although not depicted, *C. tropicalis* is capable of forming hyphal filaments (adapted from [20,50]).

In order to more precisely define the relationship between the yeast–filament transition and pathogenicity in non-*albicans Candida* species, Banerjee et al. have recently carried out a study using *C. tropicalis* and *C. parapsilosis tetO-UME6* strains genetically engineered to switch from yeast to filamentous form [49]. In vitro, these strains grow as yeast in the presence of Dox and as filaments in the absence of Dox as a consequence of constitutive high-level *UME6* expression [43]. Mice placed on drinking water in the presence and absence of Dox and inoculated with *C. tropicalis* and *C. parapsilosis tetO-UME6* strains showed equivalent fungal burdens at Day 1 post-infection [49]. Surprisingly, however, for both species, *UME6* expression led to a dramatic decline in fungal burden and eventually complete organ clearance at later post-infection time points. Histological analysis confirmed that *UME6* expression was driving filamentation during infection in vivo. In support of these findings, a *C. tropicalis* hyperfilamentous *nrg1*Δ/Δ mutant showed reduced organ fungal burdens and a filament-defective *hgc1*Δ/Δ mutant exhibited slightly increased fungal burdens [49]. Interestingly, comprehensive immune profiling did not reveal any significant alterations in the host immune response to *C. tropicalis* and *C. parapsilosis UME6* expression. Instead, whole-genome transcriptional profiling experiments demonstrated that while, as expected, several genes involved in the physical process of generating filaments were induced, other genes involved in key virulence-related processes were significantly down-regulated [49]. These genes included those encoding secreted aspartyl proteases and a phospholipase, involved in host cell degradation, iron regulators, as well as genes involved in combatting host oxidative and nitrosative stress. These findings were consistent with a previous study suggesting that proteinases are not involved in the ability of *C. tropicalis* to promote tissue damage in a reconstituted model of the human oral epithelium [45]. Interestingly, unlike the situation in *C. albicans* where genes important for driving the physical process of filamentation are co-expressed with those involved in a variety of other virulence-related processes, in at least two non-*albicans Candida* species this does not appear to be the case. Instead, certain genes likely to be associated with pathogenesis processes appear to be down-regulated, rather than up-regulated, during filamentation which may, in part, explain the observed reduced pathogenicity (Figure 1). 

Finally, recent work has shed new light on the relationship between filamentation and pathogenicity in the emerging human fungal pathogen *C. auris*. Until recently, this species was not believed to undergo filamentation. However, Kim et al. have demonstrated that *C. auris* can transition from yeast to filamentous cells in response to cell cycle arrest or depletion of the key molecular chaperone Hsp90, but not in response to conditions that normally drive *C. albicans* filamentation [51]. This study further highlights the differences between filamentation in *C. albicans* vs. NACS. Interestingly, another recent report demonstrated that *C. auris* can undergo a heritable phenotypic switch from yeast to both filamentation-competent yeast as well as filamentous cells upon passage through a mammalian host in vivo [52]. *C. auris* yeast and filamentous cells showed equivalent fungal burdens in an intraperitoneal infection. Equivalent fungal burdens were also observed in a systemic infection in kidneys, livers, and spleens, although higher burdens were observed for filamentous vs. yeast cells in brains and lungs. Filamentous cells also appeared to be more invasive in a skin infection model. One caveat to these experiments, however, is that they were carried out using high *C. auris* inoculum sizes, which could have affected the experimental outcome (see below). Interestingly, transcriptional profiling revealed that while orthologs of several *C. albicans* filament-specific genes were induced during *C. auris* filamentation, orthologs of two key positive transcriptional regulators of *C. albicans* filamentation were actually down-regulated. These findings suggest that if there is a positive correlation between filamentation and pathogenicity in *C. auris*, it appears to occur in a niche-specific manner.

## 5. Evolutionary Differences in the Relationship between Morphology and Pathogenicity in *C. albicans* vs. Non-*albicans Candida* Species

The recent finding that enhanced filamentation is associated with reduced, rather than increased, pathogenicity of *C. tropicalis* and *C. parapsilosis* suggests that there are fundamental differences in the evolutionary relationship between morphology and pathogenicity when comparing *C. albicans* vs. NACS [49]. Importantly, these results challenge conventional views that NACS are less pathogenic than *C. albicans* because they do not filament as well and instead suggest that other virulence properties, besides filamentation, play a more prominent role in the pathogenesis of certain NACS. In this respect, certain NACS appear to be better adapted to survive in the host when growing in the yeast form. How did such important evolutionary differences in the relationship between morphology and pathogenicity arise among *Candida* species? Although a definitive answer to this question is lacking, transcriptional profiling experiments suggest that while genes important for filamentation per se are up-regulated in both *C. albicans* and NACS, several genes associated with other key virulence properties are significantly down-regulated in certain NACS [49]. In contrast, in *C. albicans*, genes important for filamentation are coordinately expressed with those involved in a wide variety of virulence-related processes, including adhesion and the secretion of degradative enzymes [53,54]. This coordinate expression, in turn, may provide *C. albicans* with a distinct evolutionary advantage during infection of the host, and could partially explain why approximately 50% of all *Candida* infections can be attributed to this species [12] (Figure 1). Coordinate expression of genes important for both filamentation and other virulence-related processes, however, may be more of an exception rather than the rule among *Candida* species. Another important question that arises is why have NACS retained the ability to filament if filamentation confers an evolutionary disadvantage during infection? One possible explanation is that filamentation may represent a vestigial nutrient scavenging response for these species. Alternatively, because *C. tropicalis* filaments are more readily apparent during mouse systemic infections at higher inoculum sizes [55], they may play a more prominent role in pathogenicity under these conditions. However, most naturally occurring infections in the clinic result from much lower inoculum sizes at which *C. tropicalis* is typically found in the yeast form [55]. In this case, a threshold level of *C. tropicalis* cells may need to be reached during infection, at which point filaments might promote pathogenesis rather than leading to organ clearance. While the role and purpose of filamentation in NACS remains unclear at this point, the recent findings of Banerjee et al. strongly suggest that not everything that has been learned about virulence properties of *C. albicans* can simply be applied to the NACS, but rather instead NACS need to be studied in their own right [49]. In addition, these findings raise many new and important questions for further study. Can reduced pathogenicity of NACS be attributed to lowered expression of particular genes or gene classes? Are filaments generated by NACS vs. *C. albicans* inherently less fit during infection in vivo and, if so, why? Do filamentous NACS strains have the potential to serve as live attenuated vaccines for protection against infections by multiple *Candida* species? Continued research in this area is likely to shed more light on the complex relationship between morphology and pathogenicity in *Candida* species, which may eventually provide information leading to the development of more effective broad-spectrum antifungal strategies.
